# Starter Culture‐Induced Fermentation Reveals Genotype‐Driven Variability in the Composition and Quality of Brazilian Amazon Forastero Cocoa Beans

**DOI:** 10.1111/1750-3841.70908

**Published:** 2026-02-13

**Authors:** Giulia Victória Silva Lima, Anne Suellen Oliveira Pinto, Maria Glaucilene dos Santos Correia, Marcos Paulo Meireles Filho, Andre da Luz de Freitas, Patrícia Oliveira Santos, Hervé Rogez

**Affiliations:** ^1^ Centre for Valorization of Amazonian Bioactive Compounds (CVACBA) Universidade Federal do Pará (UFPA), Guamá Science and Technology Park Belém Pará Brazil; ^2^ Department of Agroindustry Instituto Federal de Ciência e Tecnologia do Pará (IFPA) Castanhal Pará Brazil

## Abstract

Cocoa (*Theobroma cacao* L.) is a crop of major economic importance, with Brazil ranking sixth in global production and Pará leading national output. Bean quality is strongly influenced by genetic diversity and postharvest processing, particularly fermentation. To minimize batch heterogeneity, this study evaluated 18 Forastero genotypes from the Brazilian Amazon under induced fermentation with selected starter cultures. Pods were collected from the CEPLAC germplasm bank (Medicilândia, Pará). Small‐scale fermentations (800 g, 144 h) were conducted using *Pichia kudriavzevii*, *Lactiplantibacillus plantarum*, and *Acetobacter pasteurianus*, followed by sun drying. Beans were analyzed for physical traits, cut test, pH, fermentation index (FI), total polyphenols (Folin–Ciocalteu), sugars, and organic acids (UHPLC). After fermentation, the average pH reached 4.97, and the moisture decreased below 8 g/100 g, ensuring stability. The FI increased from 0.62 to 1.02, indicating adequate fermentation except for CAB324, MA11, MA15, and IMC67. Polyphenols decreased significantly in nine genotypes, ranging from 11.32 to 24.59 g GAE/100 g DM. Sucrose was almost completely hydrolyzed, resulting in higher fructose and variable glucose levels. Bean sizes varied from standard to very small. Principal component analysis grouped genotypes by chemical traits: CCN51 (larger beans, high fructose/glucose), PA195/PA121 (greater acidity), and IMC67 (pH 5.85, residual sucrose, violet cotyledons) showed incomplete fermentation. The results demonstrate that, even under controlled starter‐culture fermentation, genetic background strongly determines the chemical composition and quality of Brazilian Amazon cocoa beans.

**Practical Applications:**

This study provides insights for cocoa producers and chocolate manufacturers to optimize fermentation according to genotype‐specific responses. By using starter cultures and understanding genetic influences on bean chemistry, producers can achieve more consistent quality, supporting the development of traceable and high‐value Amazonian cocoa products.

## 1 Introduction

Cocoa beans (*Theobroma cacao* L.) are the primary raw material for the chocolate and confectionery industries, representing a commodity of major economic relevance. Brazil currently ranks sixth among the world's largest cocoa producers, with a production of 296,145 tons in 2023 (International Cocoa Organization 2024). Among the producing states, Pará, located in the Amazon region, stands out as the leading national producer, accounting for approximately 47% of Brazil's output (Instituto Brasileiro de Geografia e Estatística 2024).

Although Forastero cocoa beans, native to the Amazon, constitute the basis of bulk cocoa and account for approximately 80% of global production (Castro‐Alayo et al. 2019), evidence indicates that cocoa produced in Pará exhibits distinctive characteristics, such as a unique aromatic profile and high concentrations of bioactive compounds, making it a promising raw material for the premium chocolate market (Collin et al. 2023; Silva et al. 2024; Lima et al. 2025).

However, genetic variation among cocoa trees, as well as differences in postharvest processing methods, can significantly influence these properties and, consequently, the final quality of the beans (Febrianto and Zhu 2019; dos Santos et al. 2024; Lima et al. 2024). In practice, however, most cocoa plantations are composed of hybrids derived from different varieties (Kongor et al. 2016; Doaré et al. 2020; Lima et al. 2025).

In Pará state, approximately 12 to 20 hybrid genotypes, resistant and highly productive, developed with the support of institutions such as the Executive Commission of the Cocoa Farming Plan (Comissão Executiva do Plano da Lavoura Cacaueira [CEPLAC]), are cultivated and processed together to mitigate losses caused by pests and diseases (Lopes et al. 2011; Pinto et al. 2024). Although this strategy provides agronomic advantages, it may also result in heterogeneous batches (Moreira et al. 2013).

The main step in postharvest processing is fermentation, characterized by a spontaneous and dynamic succession. Yeasts act predominantly in the initial anaerobic phase, fermenting pulp sugars into ethanol and producing compounds such as CO_2_, organic acids, and other metabolites that contribute to pulp degradation. Lactic acid bacteria (LAB) convert sugars and part of the ethanol into lactic acid, acetic acid, and mannitol, thereby lowering the pH. In the final stage, acetic acid bacteria (AAB) oxidize ethanol into acetic acid, an exothermic reaction that increases the temperature of the fermenting mass and leads to embryo death (De Vuyst and Leroy 2020).

Although fermentation takes place in the pulp surrounding the seeds and not directly in the cotyledons, the process involves the diffusion of compounds between the cotyledons and the fermenting environment, promoting chemical and physical transformations that are essential for cocoa quality development (De Vuyst et al. 2010). Microbial metabolites that diffuse into the seeds activate endogenous enzymatic reactions, such as the degradation of polyphenols and the formation of precursor molecules of flavor and aroma (Chagas Junior et al. 2021).

The microbial communities associated with cocoa fermentation vary across different geographic regions, and both the duration and conditions of the process are influenced by local agricultural practices (Lima et al. 2024). This scenario has motivated the exploration of starter cultures to standardize cocoa bean fermentation processes (Lefeber et al. 2012; Pereira et al. 2012; Batista et al. 2015; Visintin et al. 2017). In general, an effective starter culture can guide cocoa fermentation and should include at least one strain from each major microbial group.

This study investigates the impact of starter culture‐induced fermentation (*Pichia kudriavzevii* LPB 06, *Lactiplantibacillus plantarum* L36, and *Acetobacter pasteurianus* A06) on the physicochemical properties of cocoa beans from 18 Amazonian Forastero genotypes, focusing on sugars, organic acids, polyphenols, and overall fermentation quality. The hypothesis was that small‐scale fermentation with defined starter cultures could reduce postharvest variability and better reveal genotype‐driven biochemical differences.

## 2 Materials and Methods

### 2.1 Cocoa Genotypes

Fruits from 18 Forastero cocoa genotypes (CCN51, P7, CA6, PA121, PA195, PA169, RB40, RB36, CAB499, CAB324, CAB208, CAB270, CAB214, MA11, MA15, IMC67, MO1, and BE10) were collected from the CEPLAC germplasm bank (Medicilândia, Pará) during the 2020 harvest (Figure S1). The cocoa genotypes investigated in this study are established improved genotypes maintained in ex situ CEPLAC germplasm collections and/or registered in the International Cocoa Germplasm Database (ICGD). General background information on these genotypes is available in (Lima et al. 2025) and (Pinto et al. 2024), while detailed genetic and agronomic information can be accessed through the online database of the ICGD (Turnbull 2026). About 70 pods per genotype were collected from multiple trees at a similar maturity stage. The pods were surface‐disinfected (sodium hypochlorite 2%, 15 min), opened under a laminar flow cabinet, and pooled by genotype. Subsequently, 1 kg of pulp‐covered seeds was collected and stored in sterile plastic bags.

### 2.2 Identification and Selection of Microorganisms for Starter Cultures

The yeast *Pichia kudriavzevii* LPB 06 was previously isolated from a spontaneous cocoa fermentation in the Brazilian Amazon and used as a starter culture (Pereira et al. 2017). Lactic acid bacteria (LAB) and acetic acid bacteria (AAB) were isolated from cocoa seeds obtained from a spontaneous fermentation in Tomé‐Açu (Pará, Brazil). LAB isolates were obtained on De Man, Rogosa, and Sharpe agar (MRS, Merck), while AAB isolates were obtained on GYC agar (glucose 50 g/L, yeast extract 10 g/L, calcium carbonate 20 g/L, and agar 15 g/L). Plates were incubated at 36°C for 48 h. Distinct colonies from triplicate plates were purified and preliminarily characterized based on colony morphology, Gram staining, and catalase and oxidase tests (Pereira et al. 2012).

The molecular identification of the bacterial isolates was based on 16S rRNA gene sequencing. DNA was extracted with the ZR Fungal/Bacterial DNA MicroPrep Kit (D6007), amplified using primers 27‐F (5′‐AGAGTTTGATCCTGGCTCAG‐3′) and 1512‐R (5′‐CGGCTACCTTGTTACGACT‐3′) in a Veriti Thermal Cycler (Applied Biosystems), and purified using Agencourt AMPure XP (Beckman Coulter, Pasadena, CA). Sequencing was performed with the BigDye Terminator v3.1 Cycle Sequencing Kit (Applied, Cleveland, OH) on an ABI 3500 Genetic Analyzer (Applied Biosystems, Foster City, CA), and the sequences were aligned in BioEdit 7.7 and identified by BLAST against GenBank (National Center for Biotechnology Information, Bethesda, MD).

Following identification, LAB strains were screened for β‐glucosidase activity on TSA supplemented with esculin (0.1%) and ferric ammonium citrate (0.05%), according to Liang et al. (2001). Colonies were inoculated at the center of the plates, and β‐glucosidase activity was confirmed by the formation of black–brown halos after 12–24 h of incubation. Based on this screening, *L. plantarum* L36 was selected as the LAB starter culture. The strain *A. pasteurianus* A06 was selected from the same set of isolates obtained from the spontaneous fermentation in Tomé‐Açu and used as the AAB starter culture.

### 2.3 Preparation of Starter Culture Inoculum

*P. kudriavzevii* LPB 06 was cultivated in YPD broth (yeast extract 10 g/L, peptone 10 g/L, and dextrose 20 g/L). *L. plantarum* L36 was cultured in MRS broth, and *A. pasteurianus* A06 in GYP broth (glucose 50 g/L, yeast extract 10 g/L, peptone 30 g/L). All cultures were incubated at 36°C for 24 h. Cells were harvested by centrifugation (4000 rpm, 10 min) and resuspended in 8 mL of peptone solution (1 g/L). The yeast cell concentration was adjusted to 10^8^ cells/mL by direct counting using a Neubauer chamber (Brand GmbH, Germany) under a light microscope, whereas for LAB and AAB, the concentration was adjusted by optical density (OD) at 600 nm, with OD = 0.5 for LAB and OD = 0.2 for AAB, according to McFarland standards.

### 2.4 Starter Culture‐Induced Fermentations

#### 2.4.1 Fermentation System Setup

**FIGURE 1:**
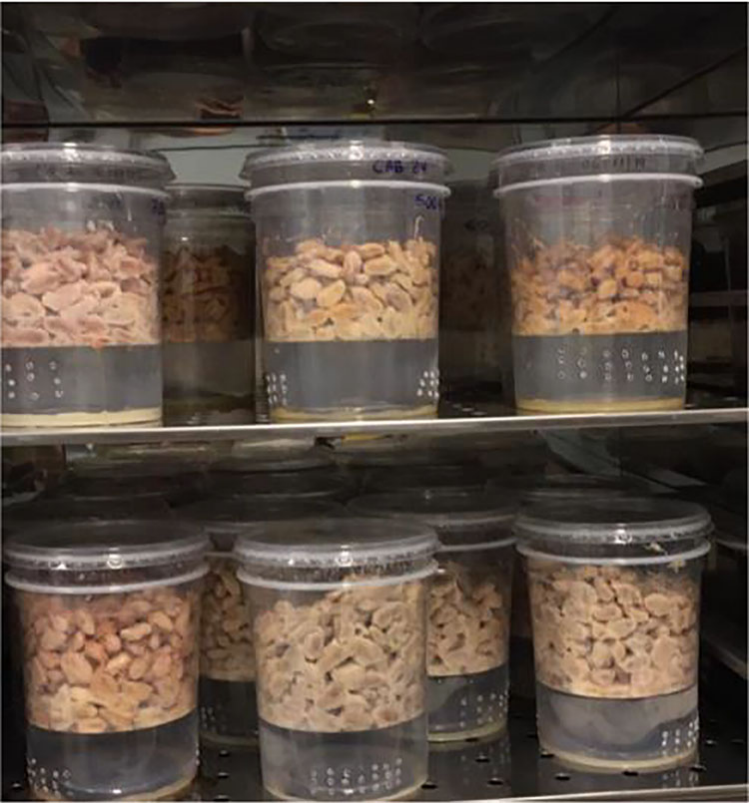
Small‐scale systems for induced cocoa fermentation.

For the induced fermentation experiments, two coupled transparent polypropylene containers were used: one with a capacity of 1.5 L and another with 1.8 L (Figure 1). The inner 1.5 L container, designed to hold the cocoa seeds, was perforated at the bottom to allow the drainage of fermentation liquor (“sweatings”). The outer 1.8 L container collected the drained liquor and helped maintain an initial anaerobic environment. After the first 48 h of fermentation, the outer container was also perforated to facilitate the transition to an aerobic environment.

#### 2.4.2 Inoculation and Fermentation Management

Fermentations were performed according to the methodology proposed by Pereira et al. (2012), with adaptations. Approximately 800 g of seeds from each cocoa genotype were placed into the fermentation systems (see section 2.4.1), inoculated with 10% (v/v) active cell suspension (10^8^ cells/g). Yeast and LAB cultures were added at the start, and containers were sealed to restrict gas exchange with the external environment and create anaerobic conditions (John et al. 2019). After 48 h, AAB was introduced to simulate the spontaneous colonization, and lids were replaced with perforated ones to allow gas exchange. Beans were turned every 24 h, except during the first 48 h.

The fermentation systems were maintained in a bacteriological incubator (model LUCA 81/180, Lucadema, São José do Rio Preto, SP, Brazil). Temperature followed a gradual increase throughout fermentation and was monitored using a thermometer inserted into the fermenting mass to ensure appropriate internal conditions. Seeds were initially maintained at 28°C, raised to 30°C after 12 h, 32°C after 24 h, 35°C at 36 h, 38°C at 48 h, 42°C at 60 h, 46°C at 72 h, and finally reached 48°C at 84 h, which was maintained until the end of the process (144 h). Subsequently, beans were sun‐dried for 5 days until reaching a moisture content below 8 g/100 g (International Organization for Standardization 2017).

### 2.5 Physical and Physicochemical Analyses of Cocoa Beans

#### 2.5.1 Bean Size, Weight, and Count

The dimensions of fermented and dried beans were measured using a caliper, and results were expressed as the average of 20 randomly selected beans from each genotype. Measurements included length, width, and thickness. Average bean weight and the number of beans per 100 g were determined using an analytical balance (AUY220, Shimadzu).

#### 2.5.2 Cut Test

The cut test consisted of a longitudinal cut of 30 fermented and dried beans, randomly selected to visually assess the color and compartmentalization of the cotyledons. Results were expressed as percentages, categorizing beans by color (brown, partially brown, or purple) and compartmentalization (compartmentalized or uncompartmentalized) (International Organization for Standardization 2017).

#### 2.5.3 Moisture and pH

Moisture content was determined gravimetrically according to the Association of Official Analytical Collaboration (AOAC) method 931.04 (AOAC International 2023). To measure the internal pH of the beans, samples were homogenized in distilled water at a 1:10 (w/v) ratio, filtered, and the pH was determined using a digital pH meter (model PHS 3BW, Bel Engineering) (Senanayake et al. 1997).

### 2.6 Spectrophotometric Analyses

#### 2.6.1 Fermentation Index

Approximately 100 mg of ground cocoa cotyledons were extracted with 10 mL of a methanol:HCl solution (97:3, v/v), refrigerated at 8°C for 18 h, and then filtered. The supernatant was analyzed using a spectrophotometer, and the FI was calculated as the ratio between absorbance readings at 460 and 530 nm (Gourieva and Tserrevitinov 1979).

#### 2.6.2 Total Polyphenol Content

Ground cocoa cotyledons were defatted with diethyl ether at a 1:5 (w/v) ratio at room temperature under agitation for 1 min (Lima et al. 2025). Phenolic compounds were extracted from 100 mg of defatted cocoa powder with 10 mL acetone:water:acetic acid (70:29.5:0.5 v/v/v) following the AOAC method 2017.13 (AOAC International 2023). Extracts were vortexed (1 min), sonicated (5 min, 50°C), centrifuged (5 min, 3000 rpm), and diluted (1:10). For each assay, 1500 µL of water, 100 µL of the diluted extract, and 100 µL of Folin–Ciocalteu reagent (2 mol/L) were mixed. After 6 min, 300 µL of a 20% calcium carbonate solution was added. The reaction was kept in the dark for 2 h for color development, and absorbance was measured at 765 nm using a spectrophotometer (Biotrak, Molecular Devices, Sunnyvale, CA). Quantification was performed by comparison with a calibration curve prepared using gallic acid as the standard. Analyses were performed in triplicate, and results were expressed as grams of gallic acid equivalents per 100 g of dry matter (g GAE/100 g DM).

### 2.7 Chromatographic Analyses

About 100 mg of deffated cocoa powder was solubilized in 1.9 mL of ultrapure water, vortexed (1 min), heated in a water bath (85°C, 20 min), cooled (ice bath, 10 min), and centrifuged (2500 rpm, 10 min, 25°C). The supernatant was filtered through a 0.22 µm PVDF syringe filter (Analítica, São Paulo, SP, Brazil), and 10 µL was injected into an Aminex HPX‐87H column (300 × 7.8 mm, Bio‐Rad Laboratories, Hercules, CA, USA).

Sugars (D‐(+)‐glucose, D‐(−)‐fructose, and sucrose) and organic acids (citric, acetic, and oxalic) were analyzed on a Thermo Scientific Ultimate 3000 UHPLC (San Jose, CA, USA) with refractive index (RI) and diode array detector (DAD) detectors. Acids were monitored at 200 nm (oxalic), 207 nm (citric), and 204 nm (acetic). Isocratic elution used ultrapure water acidified with 5 mM H_2_SO_4_ at 0.5 mL/min (Kubola et al. 2011).

Analyses were performed in triplicate. Compounds were identified by retention time against authentic standards, and concentrations were calculated from calibration curves based on standard peak areas.

### 2.8 Statistical Analyses

Data normality was assessed with the Shapiro–Wilk test. Paired *t*‐tests were applied to normally distributed variables and Wilcoxon tests to nonnormal ones, with significance set at *p* < 0.05. Physicochemical data were normalized, and principal component analysis (PCA) was performed considering all quantitative variables. All statistical analyses were conducted in R software (version 4.4.2) using the RStudio environment (RStudio Team 2023).

## 3 Results and Discussion

### 3.1 Microorganisms Selected for Starter Culture

43 isolates were obtained on MRS medium, of which 24 were Gram‐positive and tested negative for catalase and oxidase activities, consistent with LAB. 16S rRNA gene sequencing identified 19 isolates as *L. plantarum* and 5 as *Limosilactobacillus fermentum*, all showing >99% sequence identity (Table 1). From the 10 isolates obtained on GYC medium, only one strain exhibited phenotypic traits compatible with AAB (Gram‐negative, catalase‐positive, and oxidase‐negative). 16S rRNA gene analysis identified this strain as *A. pasteurianus*, with 99.92% sequence identity (Table 1).

**TABLE 1:** Lactic acid and acetic acid bacteria isolated from a spontaneous cocoa fermentation in the Brazilian Amazon, identified by 16S rRNA gene sequencing.

Isolate	Species	Identity (%)	GenBank accession	β‐glucosidase (mm)^a^
L01	*Limosilactobacillus fermentum*	99	MK246000.1	14.0 ± 0.8
L03	*Limosilactobacillus fermentum*	99	MT515869.1	—
L04	*Limosilactobacillus fermentum*	99.9	MK418594.1	—
L15	*Lactiplantibacillus plantarum*	99	MK774555.1	—
L16	*Lactiplantibacillus plantarum*	99	MT597655.1	—
L17	*Lactiplantibacillus plantarum*	99	MH656862.1	—
L18	*Lactiplantibacillus plantarum*	99.7	MW692386.1	—
L19	*Lactiplantibacillus plantarum*	100	OK272375.1	—
L20	*Lactiplantibacillus plantarum*	99	MG913360.1	—
L21	*Lactiplantibacillus plantarum*	99.9	KX426268.1	—
L22	*Lactiplantibacillus plantarum*	99	KT278847.1	—
L24	*Lactiplantibacillus plantarum*	99	KX426268.1	—
L25	*Limosilactobacillus fermentum*	99	OL375212.1	—
L27	*Lactiplantibacillus plantarum*	99.9	MK774543.1	—
L29	*Lactiplantibacillus plantarum*	99.9	MH620395.1	—
L30	*Lactiplantibacillus plantarum*	99	MT463848.1	—
L31	*Lactiplantibacillus plantarum*	98.9	OK493619.1	—
L32	*Lactiplantibacillus plantarum*	99	MH472967.1	15.0 ± 0.7
L35	*Lactiplantibacillus plantarum*	99.4	MT515894.1	—
L36	*Lactiplantibacillus plantarum*	99	MH472949.1	20.0 ± 0.6
L37	*Limosilactobacillus fermentum*	100	MT658578.1	—
L38	*Lactiplantibacillus plantarum*	99.8	KM497499.1	—
L39	*Lactiplantibacillus plantarum*	99.3	OR436372.1	18.0 ± 0.9
L40	*Lactiplantibacillus plantarum*	100	CP120642.1	—
A06	*Acetobacter pasteurianus*	99.9	KF030738.1	—

^a^Results expressed as mean ± standard deviation of the halo diameter formed around the colonies in the β‐glucosidase activity assay.

Several studies have reported that *Lacp. plantarum* and *Liml. fermentum* are consistently present during cocoa fermentation across major producing regions, including Brazil, Côte d'Ivoire, Cameroon, Ghana, and Nicaragua (Lefeber et al. 2012; Papalexandratou et al. 2013; Adler et al. 2013; Ouattara et al. 2017; Serra et al. 2019). Fructophilic species such as *Lacp. plantarum* tend to dominate in the early stages of fermentation, using fructose as the main energy source and converting it primarily into lactic acid, thereby reducing direct competition with yeasts. As yeast activity decreases, heterofermentative species such as *Liml. fermentum* become predominant, metabolizing glucose into lactic acid, acetic acid, ethanol, carbon dioxide, and/or manitol (De Vuyst and Leroy 2020).

*Lacp. plantarum* exhibits characteristics that make it highly adapted to the cocoa fermentation ecosystem, including tolerance to temperature fluctuations, ethanol presence, and acidic stress conditions (Agyirifo et al. 2019). In addition, fructophilic strains contribute to reducing residual fructose, which would otherwise prolong fermentation and favor the late proliferation of undesirable fungi (Viesser et al. 2020).

Among the 19 *Lacp. plantarum* strains, only three showed positive results for β‐glucosidase activity (Table 1). This enzyme hydrolyzes the glycosidic bonds of anthocyanins, removes sugar fractions (e.g., glucose or arabinose), releasing their corresponding aglycones, such as cyanidin. This initial step is crucial, as it facilitates the subsequent chemical or enzymatic degradation of aglycones, thereby contributing significantly to the development of the cocoa bean sensory profile, particularly during roasting (Castro‐Alayo et al. 2019).

The average halo diameter formed by isolate L36 after 24 h of incubation was significantly larger (*p* < 0.05) than that of isolates L32 and L39 (Table 1), indicating higher β‐glucosidase activity. Therefore, *Lacp. plantarum* L36 was selected to compose the starter culture, together with *P. kudriavzevii* and *A. pasteurianus* A06, the only isolate identified as an AAB.

*P. kudriavzevii* LPB 06 is widely recognized for its relevance in Brazilian cocoa fermentation due to its tolerance to acid and ethanol, as well as its contribution to flavor modulation (Pereira et al. 2017). In addition, *A. pasteurianus* is frequently found in spontaneous fermentations and is considered one of the main agents responsible for the oxidation of ethanol, lactic acid, and mannitol. This species also exhibits strong tolerance to acidity and heat, traits that are essential for cocoa fermentation (Papalexandratou et al. 2013; De Vuyst and Weckx 2016).

### 3.2 Physicochemical Characterization of Cocoa Beans Before and After Induced Fermentation

#### 3.2.1 Moisture, pH, and Fermentation Index

The results showed a significant reduction in moisture content and pH of the cocoa beans after induced fermentation and drying, along with an increase in the fermentation index (FI) (Table 2).

**TABLE 2:** Physicochemical characteristics of cocoa beans from the Brazilian Amazon before (UF) and after starter culture‐induced fermentation (*Pichia kudriavzevii* LPB 06, *Lactiplantibacillus plantarum* L36, and *Acetobacter pasteurianus* A06) and drying (F).

Genotype	Moisture (g/100 g)		pH		Fermentation index	
	UF	F	UF	F	UF	F
CCN51	32.23 ± 0.09^d^	7.09 ± 0.10^a^	6.51 ± 0.23^a^	4.93 ± 0.00^def^	0.76 ± 0.02^a^	1.06 ± 0.01^ab^
P7	28.57 ± 0.16^ghi^	5.87 ± 0.04^defg^	6.34 ± 0.08^ab^	4.86 ± 0.01^efg^	0.49 ± 0.05^f^	1.01 ± 0.04^bcd^
CA6	27.43 ± 0.46^j^	5.60 ± 0.24^fgh^	6.23 ± 0.02^ab^	5.33 ± 0.01^b^	0.56 ± 0.00^cdef^	1.06 ± 0.02^ab^
PA121	36.94 ± 0.02^b^	6.73 ± 0.05^abc^	6.37 ± 0.01^ab^	4.72 ± 0.05^fgh^	0.61 ± 0.01^bcde^	1.02 ± 0.01^abcd^
PA195	30.45 ± 0.11^f^	6.28 ± 0.25^bcde^	6.36 ± 0.08^ab^	4.61 ± 0.01^h^	0.62 ± 0.02^bcd^	1.11 ± 0.01^a^
PA169	29.27 ± 0.36^gh^	5.72 ± 0.01^efg^	6.37 ± 0.01^ab^	4.85 ± 0.01^efgh^	0.60 ± 0.00^bcde^	1.06 ± 0.02^ab^
RB40	32.16 ± 0.36^de^	4.97 ± 0.01^hi^	6.37 ± 0.04^ab^	5.12 ± 0.00^bcd^	0.65 ± 0.00^abcd^	1.02 ± 0.04^abcd^
RB36	31.27 ± 0.08^ef^	4.71 ± 0.13^i^	6.21 ± 0.00^ab^	4.69 ± 0.01^gh^	0.51 ± 0.05^ef^	1.10 ± 0.01^ab^
CAB499	28.24 ± 0.13^ij^	6.21 ± 0.14^cdef^	6.40 ± 0.19^ab^	4.71 ± 0.01^fgh^	0.66 ± 0.03^abc^	1.05 ± 0.03^abc^
CAB324	38.72 ± 0.25^a^	6.45 ± 0.07^abcd^	6.11 ± 0.17^b^	5.06 ± 0.01^cde^	0.66 ± 0.03^abc^	0.95 ± 0.02^cde^
CAB208	28.38 ± 0.18^hij^	5.03 ± 0.34^hj^	6.23 ± 0.00^ab^	4.75 ± 0.00^fgh^	0.60 ± 0.02^bcde^	1.02 ± 0.04^abcd^
CAB270	31.41 ± 0.11^de^	6.24 ± 0.08^cdef^	6.46 ±0.14^ab^	4.70 ± 0.00^fgh^	0.66 ± 0.06^abc^	1.06 ± 0.01^ab^
CAB214	31.92 ± 0.13^de^	4.96 ± 0.07^hi^	6.36 ± 0.06^ab^	5.22 ± 0.25^bc^	0.68 ± 0.02^ab^	1.05 ± 0.01^abc^
MA11	33.85 ± 0.23^c^	5.96 ± 0.04^defg^	6.31 ± 0.03^ab^	5.02 ± 0.00^cde^	0.57 ± 0.03^bcdef^	0.95 ± 0.01^de^
MA15	32.35 ± 0.45^d^	6.93 ± 0.04^ab^	6.36 ± 0.04^ab^	5.20 ± 0.01^bc^	0.60 ± 0.03^bcde^	0.94 ± 0.03^de^
IMC67	29.34± 0.18^g^	5.48 ± 0.06^gh^	6.40 ± 0.04^ab^	5.85 ± 0.01^a^	0.55 ± 0.01^def^	0.91 ± 0.04^e^
MO1	33.62 ± 0.03^c^	5.32 ± 0.33^ghi^	6.19 ± 0.06^ab^	4.71 ± 0.01^fgh^	0.67 ± 0.01^abc^	1.10 ± 0.03^ab^
BE10	26.46 ± 0.17^k^	5.75 ± 0.27^efg^	6.21 ± 0.07a^b^	5.13 ± 0.01^bcd^	0.68 ± 0.01^ab^	1.01 ± 0.01^bcd^

*Note*: Results are expressed as mean ± standard deviation. Different letters within the same column indicate significant differences according to Tukey's test (*p* < 0.05).

The average moisture content of unfermented beans was 31.25 ± 0.13 g/100 g. During drying, heat transfer to the beans promoted the evaporation of water and volatile acids, reducing moisture values to a range of 4.96 ± 0.07 g/100 g (CAB214) to 7.09 ± 0.10 g/100 g (CCN51). Moisture contents below 8 g/100 g are essential to prevent over‐fermentation and the growth of undesirable microorganisms during storage and transport, both critical factors that determine the final quality of the beans (Santander Muñoz et al. 2020).

The PA195 genotype exhibited the most acidic beans after fermentation, with a pH of 4.61 ± 0.01, whereas the IMC67 genotype showed the highest pH (5.85 ± 0.01). pH directly affects the activity of endogenous enzymes such as proteases, which are essential for protein degradation and the formation of flavor precursors. Very low pH values (4.0–4.5) may induce nonspecific proteolysis, resulting in lower concentrations of free amino acids and the formation of hydrophobic peptides. In contrast, pH values ranging from 4.5 to 5.5 favor selective protein degradation, enabling the generation of specific precursors associated with a more desirable cocoa flavor profile (Voigt et al. 2018).

The internal acidification of the beans also induces the degradation of anthocyanins, compounds responsible for the characteristic violet coloration of unfermented cocoa cotyledons. This degradation can be assessed by the FI, calculated as the ratio of absorbance at 460 nm (representing brown pigments formed during fermentation) to 530 nm (indicating residual anthocyanins). Cocoa beans with FI < 1 are considered underfermented, whereas those with FI > 1 are classified as sufficiently fermented (Romero‐Cortes et al. 2013).

Before fermentation, the beans exhibited an average FI of 0.62 ± 0.02. As anthocyanins degraded, the FI tended to increase, reaching an average value of 1.02 ± 0.01 after induced fermentation. Only the genotypes CAB324, MA11, MA15, and IMC67 showed FI < 1, which may be associated with differences in enzymatic activity among these genotypes or with the possibility that the fermentation duration was insufficient to ensure complete degradation of anthocyanin pigments (Misnawi et al. 2003).

#### 3.2.2 Total Polyphenol Content

**FIGURE 2:**
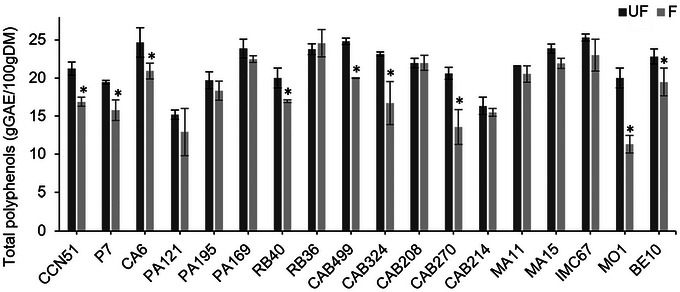
Total polyphenol content of cocoa beans from 18 Brazilian Amazon genotypes before (UF) and after starter culture‐induced fermentation (*Pichia kudriavzevii* LPB 06, *Lactiplantibacillus plantarum* L36, and *Acetobacter pasteurianus* A06) and drying (F). Results obtained using the Folin–Ciocalteu assay and expressed as grams of gallic acid equivalents per 100 grams of dry matter (g GAE/100 g DM). *Genotypes that showed statistically significant differences (*p* < 0.05) between treatments.

The results obtained from the Folin–Ciocalteu assay, presented in Figure 2, show the concentrations of total polyphenols in the cotyledons of cocoa beans before and after induced fermentation and drying.

Cocoa beans are recognized for their high polyphenol content, previously reported in the range of 12%–18% of dry weight, of which approximately 37% are flavanol monomers (epicatechin and catechin), 58% are procyanidin oligomers, and 4% are anthocyanins (Wollgast and Anklam 2000). In the Amazonian Brazilian genotypes, the initial concentrations of total polyphenols in the beans ranged from 15.20 ± 0.60 g GAE/100 g DM (PA121) to 25.30 ± 0.46 g GAE/100 g DM (IMC67).

Variations in polyphenol content are commonly attributed to factors such as growing region, fruit maturity, climatic conditions, and postharvest processing parameters, including fermentation and drying time (Febrianto and Zhu 2019; Febrianto and Zhu 2020; Lima et al. 2024). In the present study, potential biological variability among pods was minimized by collecting multiple pods from multiple trees per genotype at a similar maturity stage and pooling the material before fermentation. Moreover, all samples were obtained from a germplasm bank and subjected to uniform cultivation and processing conditions. Under these controlled conditions, the differences observed are therefore most likely associated with genotype‐related variability.

The total polyphenol concentrations in fermented cocoa beans ranged from 11.32 ± 1.17 g GAE/100 g DM (MO1) to 24.59 ± 1.80 g GAE/100 g DM (RB36). A significant reduction (*p* < 0.05) in polyphenol levels was observed in genotypes CCN51, P7, CA6, RB40, CAB499, CAB324, CAB270, MO1, and BE10 after induced fermentation and drying. In the other nine genotypes, total polyphenol levels remained stable.

In general, unfermented beans exhibit higher polyphenol concentrations, which are associated with their characteristic astringency (Aprotosoaie et al. 2016). During fermentation, the breakdown of subcellular structures in the cotyledons leads to the subsequent oxidation of polyphenols by polyphenol oxidases, generating reactive quinones. These quinones can irreversibly bind to nitrogen‐containing compounds, such as amino acids and proteins, resulting in the formation of insoluble condensed tannins (brown pigments) and a reduction in bitterness and astringency (De Vuyst and Weckx 2016).

The extent of these changes depends on enzymatic activity, which generally increases with fruit ripening but is also influenced by processing conditions (Calvo et al. 2021). In addition to oxidative reactions, part of the observed reduction can be attributed to the diffusion of polyphenols into the pulp and fermentation exudate during fermentation and drying (Santander Muñoz et al. 2020).

In genotypes where no decrease in total polyphenol concentration was observed, this outcome may be linked to the limitations of the Folin–Ciocalteu assay, which measures the cumulative content of reducing substances. Thus, besides native polyphenols, the method also detects modified or newly formed compounds during fermentation, as well as nonphenolic reducers (e.g., reducing sugars, ascorbic acid, and early Maillard reaction products), potentially leading to an overestimation of the measured values (Pérez et al. 2023).

In contrast, the application of a targeted metabolomic approach using UPLC–MS/MS on the same Amazonian cocoa genotypes evaluated in this study, but subjected to on‐farm induced fermentation, revealed a significant reduction in most phenolic compounds, with the exception of protocatechuic acid (Lima et al. 2025).

The dynamics of polyphenols during cocoa fermentation are more complex than initially assumed. Indeed, small‐scale induced fermentations have previously reported increases in polyphenol levels after processing (Evina et al. 2016; John et al. 2019), a result partially attributed to excess acidity, which may promote the retention or even release of bound forms of polyphenols.

Although high concentrations of polyphenols may negatively affect the beans from a sensory perspective, their preservation can also represent an advantage due to the potential health benefits associated with cocoa polyphenols, including antioxidant, antiproliferative, antimutagenic, and chemoprotective effects (Martin and Ramos 2021). The high antioxidant capacity of Amazonian cocoa beans has already been described (Lima et al. 2024).

Considering that both the concentration and the composition of polyphenols influence the bioactivity of cocoa and its derived products, further research is needed to elucidate the impact of fermentation processes on the specific profiles of these compounds.

#### 3.2.3 Sugars

**FIGURE 3:**
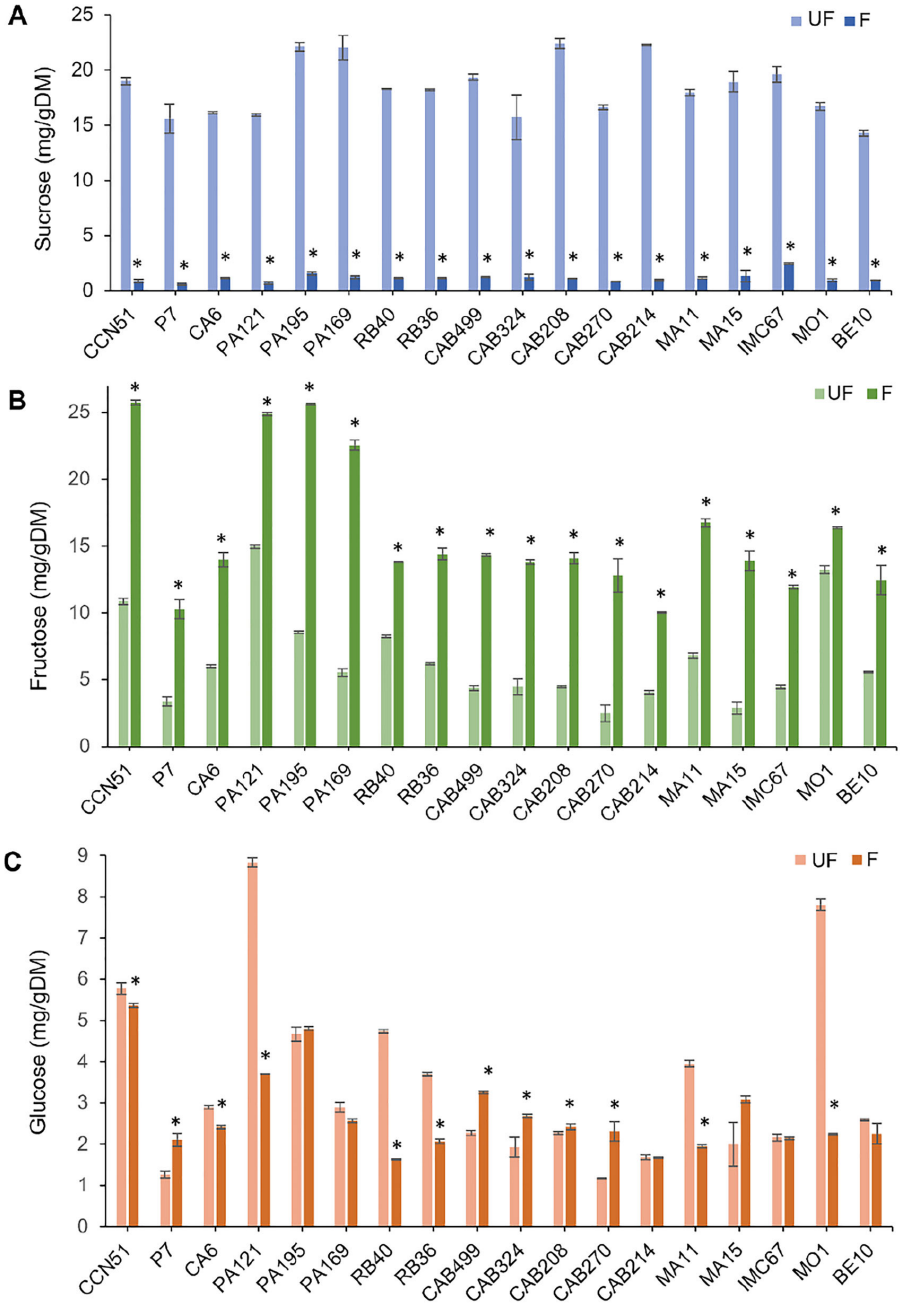
Concentration of reducing sugars in the cotyledons of cocoa beans from 18 Brazilian Amazon genotypes before (UF) and after starter culture‐induced fermentation (*Pichia kudriavzevii* LPB 06, *Lactiplantibacillus plantarum* L36, and *Acetobacter pasteurianus* A06) and drying (F): (A) sucrose; (B) fructose; (C) glucose. Results are expressed in milligrams per gram of dry matter (mg/g DM). *Genotypes that showed statistically significant differences (*p* < 0.05) between treatments.

The initial concentrations of sugars in cocoa cotyledons varied widely among genotypes. In unfermented beans, sucrose was the predominant sugar (Figure 3A), ranging from 14.30 ± 0.28 mg/g DM (BE10) to 22.40 ± 0.45 mg/g DM (CAB208). Fructose was the second most abundant sugar (Figure 3B), followed by glucose (Figure 3C). The genotype PA121 showed the highest fructose (14.95 ± 0.15 mg/g DM) and glucose (8.83 ± 0.10 mg/g DM) contents, while CAB270 displayed the lowest concentrations (2.50 ± 0.60 mg/g DM and 1.17 ± 0.01 mg/g DM, respectively).

After induced fermentation, changes in sugar concentrations followed similar trends across genotypes. Fructose became the predominant sugar, with values ranging from 25.72 ± 0.18 mg/g DM (CCN51) to 10.03 ± 0.04 mg/g DM (CAB214). In contrast, sucrose was significantly reduced in all genotypes, reaching concentrations between 2.46 ± 0.06 mg/g DM (IMC67) and 0.61 ± 0.05 mg/g DM (PA121). Glucose concentrations were more variable, ranging from 5.36 ± 0.05 mg/g DM (CCN51) to 1.64 ± 0.00 mg/g DM (RB40).

Previous studies confirm that sucrose is the predominant sugar in unfermented cocoa beans and that, during fermentation, sucrose is hydrolyzed, leading to significant increases in fructose and glucose (Ho et al. 2014; Niemenak et al. 2020). This process is attributed to the activity of invertase enzymes, which cleave sucrose, as well as acid hydrolysis promoted by the warm and acidic conditions in the cotyledons (Akoa et al. 2023).

After induced fermentation, sucrose was almost completely hydrolyzed in the Amazonian cocoa beans, resulting in an average increase of 9.51 mg/g DM in fructose. However, final glucose concentrations were lower than expected based on the hydrolysis of the initial sucrose and only increased significantly in genotypes P7, CAB499, CAB324, CAB208, and CAB270.

The discrepancy between fructose and glucose contents may be associated with the preferential metabolism of glucose or its subsequent polymerization after sucrose hydrolysis, phenomena already described during cocoa fermentation (De Brito et al. 2001). This behavior may also be explained by glucose diffusion into the pulp and subsequent microbial consumption, as well as its conversion into other compounds during drying (Ho et al. 2014).

The differences observed in sugar concentrations suggest that intrinsic factors of each genotype, such as chemical composition and enzymatic activities, play a determining role (Akoa et al. 2023; Moretti et al. 2023). Similar behaviors were reported by Niemenak et al. (2020) when simulating the fermentation process with different cocoa varieties. Even when all varieties were subjected to the same treatments, differences in sugar degradation percentages were attributed to genetic variation.

Changes in reducing sugar concentrations are critical for cocoa flavor development, since the carbonyl groups of these sugars participate in Maillard reactions with amino acids during roasting—an essential step for the formation of characteristic flavor and aroma notes (Afoakwa et al. 2008). The greater the availability of reducing sugars, the higher the likelihood of these reactions (Moretti et al. 2023).

Although roasting was not addressed in this study, it is during this process that a marked decrease in reducing sugars is observed, particularly fructose, due to its higher initial content and the high reactivity of ketoses in Maillard reactions. Glucose, however, shows more variable behavior, with reports ranging from complete consumption to the maintenance of its levels after roasting (De Brito et al. 2001).

#### 3.2.4 Organic Acids

**FIGURE 4:**
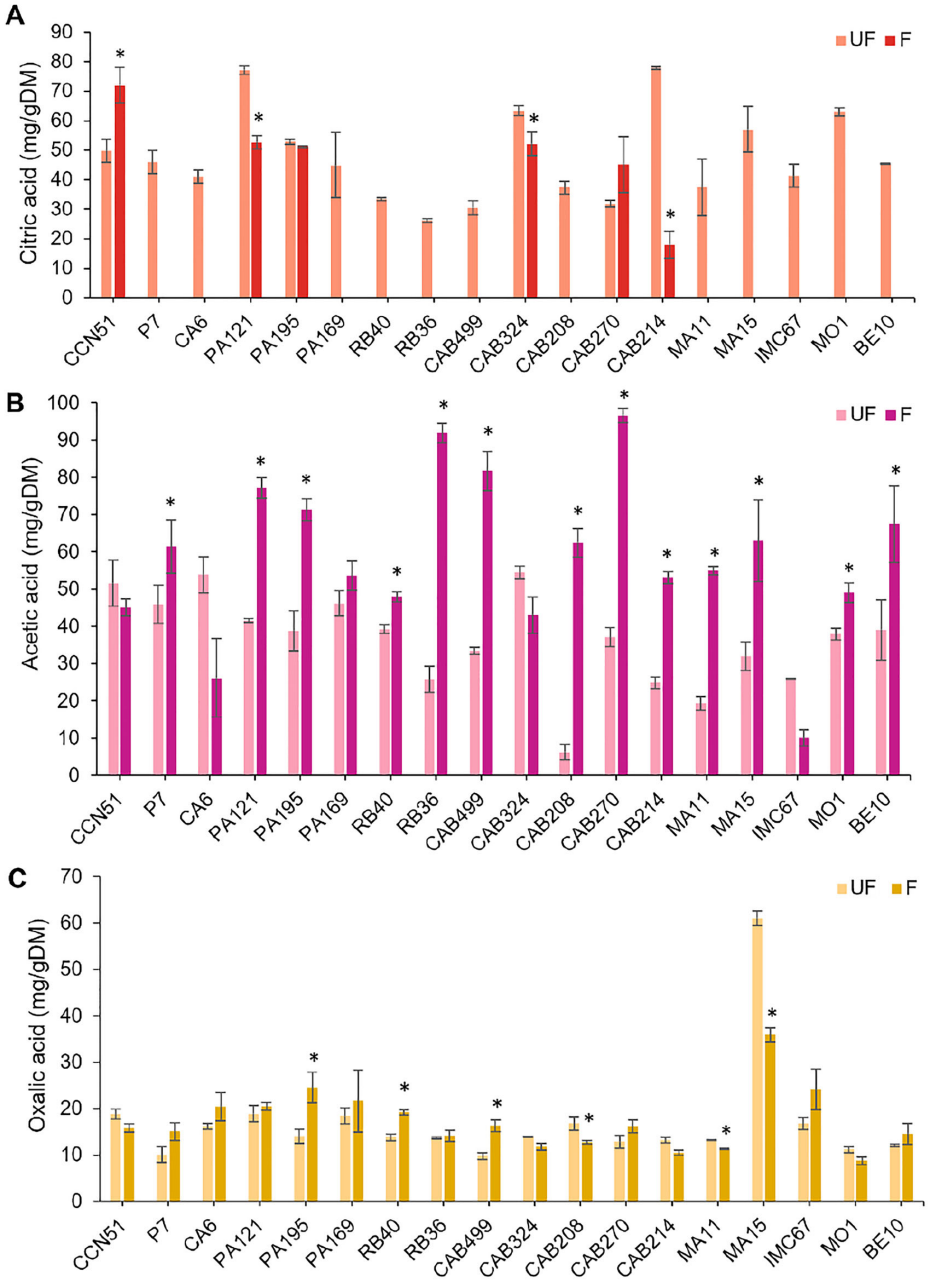
Organic acid concentrations in the cotyledons of cocoa beans from 18 Brazilian Amazon genotypes, before (UF) and after starter culture‐induced fermentation (*Pichia kudriavzevii* LPB 06, *Lactiplantibacillus plantarum* L36, and *Acetobacter pasteurianus* A06) and drying (F): (A) citric acid; (B) acetic acid; (C) oxalic acid. Results are expressed in milligrams per gram of dry matter (mg/g DM). *Genotypes that showed statistically significant differences (*p* < 0.05) between treatments.

Citric acid was the predominant organic acid in the cotyledons before fermentation (Figure 4A), with concentrations ranging from 26.20 ± 0.52 mg/g DM (RB36) to 77.84 ± 0.35 mg/g DM (CAB214). After induced fermentation, a significant reduction in citric acid concentrations was observed in most genotypes, becoming nonquantifiable in fermented beans of P7, CA6, PA169, RB40, RB36, CAB499, CAB208, MA11, MA15, IMC67, MO1, and BE10.

Citric acid is the main organic acid found in unfermented cocoa and serves as a substrate for the growth of yeasts and LAB during the early stages of fermentation. Citric acid is metabolized via oxaloacetic acid into acetic and pyruvic acids, the latter being further converted into final products of pyruvate metabolism (Camu et al. 2007). However, previous studies indicate that changes in citric acid concentrations in the pulp are not directly related to those observed within the cotyledons, suggesting that citric acid may undergo independent metabolism in the cotyledons (De Vuyst et al. 2010; Lefeber et al. 2010).

The genotypes CAB270 and PA195 maintained constant levels of citric acid after fermentation, whereas CCN51 was the only one to show an increase. Both reductions and increases in citric acid have been previously reported in small‐scale induced fermentations (Ho et al. 2014). From a sensory perspective, since citric acid is a nonvolatile compound, its persistence or accumulation in the cotyledons may contribute to the perception of residual acidity in fermented beans.

Acetic acid, detected in concentrations ranging from 6.26 ± 2.09 mg/g DM (CAB208) to 54.47 ± 1.78 mg/g DM (CAB324) before fermentation (Figure 4B), showed a significant increase in most fermented beans, reaching values between 10.07 ± 2.17 mg/g DM (IMC67) and 96.63 ± 1.97 mg/g DM (CAB270).

Acetic acid is mainly produced during fermentation through ethanol oxidation by AAB. In addition, heterofermentative LAB species, such as *Lacp. plantarum*, may also contribute to acetic acid production, although at lower concentrations (De Vuyst et al. 2010). The diffusion of acetic acid into the cotyledons plays a crucial role in cocoa fermentation, as acidification, combined with the temperature rise caused by the exothermic reactions of AAB metabolism, destabilizes subcellular structures and leads to embryo death, preventing germination (Schwan and Wheals 2004; De Vuyst and Weckx 2016).

Oxalic acid concentrations remained virtually unchanged after fermentation in most genotypes (Figure 4C). Significant changes were detected only in PA195, RB40, CAB499, CAB208, MA11, and MA15. Beans from MA15 showed the highest levels, decreasing from 61.08 ± 1.59 mg/g DM before fermentation to 35.94 ± 1.46 mg/g DM after the process, while the average of the other genotypes ranged from 14.35 ± 0.94 mg/g DM (UF) to 16.34 ± 1.79 mg/g DM (F), respectively. For comparison, concentrations of 7–8 mg/g of oxalic acid have been reported in unfermented Trinitario beans, remaining stable during induced fermentations (Ho et al. 2014).

Like other weak acids, such as succinic and malic acids, oxalic acid can be produced by yeast metabolism. These compounds play an important role in pH regulation due to their buffering capacity, helping to reduce abrupt fluctuations during fermentation (Schwan and Wheals 2004).

Organic acids are fundamental to the development of cocoa's sensory attributes. In addition to directly influencing bean acidity, pH modulation (see Section 3.2.1) affects the activity of enzymes such as proteases and invertases, which are essential for protein and carbohydrate degradation and for generating biochemical precursors of flavor and aroma (Santander Muñoz et al. 2020).

### 3.3 Physical Characteristics of Fermented and Dried Cocoa Beans

#### 3.3.1 Size, Weight, and Bean Count

The physical characterization of cocoa beans is essential for improving fermentation, drying, and processing, as well as meeting market requirements related to size and quality (International Cocoa Organization 2023). The weight, dimensions (length, width, and thickness), and the number of beans per 100 g from 18 Amazonian Forastero cocoa genotypes after induced fermentation and drying were evaluated. Results are shown in Table 3.

**TABLE 3:** Physical characteristics of fermented and dried beans from 18 cocoa (*Theobroma cacao* L.) genotypes cultivated in the Brazilian Amazon after starter culture‐induced fermentation (*Pichia kudriavzevii* LPB 06, *Lactiplantibacillus plantarum* L36, and *Acetobacter pasteurianus* A06) and drying.

Genotype	Length (mm)	Width (mm)	Thickness (mm)	Weight (g)	Count (beans/100 g)
CCN51	25.47 ± 2.00^a^	13.82 ± 0.99^a^	6.95 ± 0.89^bcd^	1.72 ± 0.26^a^	69.50 ± 0.70^i^
P7	23.65 ± 1.90^abcd^	12.53 ± 0.72^bc^	6.19 ± 0.70^defg^	1.09 ± 0.15^cdefg^	93.00 ± 1.73^fg^
CA6	23.21 ± 1.14^cde^	13.22 ± 0.91^ab^	6.26 ± 0.60^cdefg^	1.19 ± 0.19^bcdef^	85.60 ± 2.52^gh^
PA121	22.31 ± 1.56^cdef^	11.87 ± 0.69^cde^	7.06 ± 0.83^bcd^	1.08 ± 0.10^cdefg^	94.00 ± 3.00^ef^
PA195	21.51 ± 1.49^efg^	11.89 ± 0.69^cde^	8.78 ± 1.42^a^	1.22 ± 0.22^bcde^	88.00 ± 3.61^fgh^
PA169	20.98 ± 1.59^fg^	12.09 ± 0.95^cde^	7.30 ± 0.87^b^	1.04 ± 0.15^defg^	103.3 ± 3.51^de^
RB40	21.09 ± 1.63^fg^	10.81 ± 0.81^fgh^	5.58 ± 0.81^fg^	0.73 ± 0.10^ij^	127.0 ± 1.00^b^
RB36	21.88 ± 1.56^defg^	10.53 ± 0.86^gh^	7.38 ± 0.87^b^	0.95 ± 01.0^fghi^	107.6 ± 2.52^cd^
CAB499	20.33 ± 2.14^fg^	11.16 ± 0.99^efg^	5.55 ± 0.66^fg^	0.89 ± 0.15^ghij^	108.5 ± 4.44^c^
CAB324	23.92 ± 1.68^abcd^	12.22 ± 0.89^cd^	5.73 ± 0.80^efg^	0.76 ± 0.17^hij^	118.0 ± 3.00^b^
CAB208	20.75 ± 1.34^fg^	10.08 ± 0.49^h^	5.29 ± 0.44^g^	0.71 ± 0.12^ij^	142.5 ± 2.18^a^
CAB270	24.02 ± 1.31^abc^	11.57 ± 0.54^cdef^	6.82 ± 0.45^bcd^	1.00 ± 0.21^efgh^	97.67 ± 6.81^de^
CAB214	19.95 ± 1.60^g^	10.12 ± 0.51^h^	5.35 ± 0.58^g^	0.67 ± 0.12^j^	149.3 ± 0.58^a^
MA11	23.58 ± 1.24^abcd^	12.36 ± 0.64^bcd^	7.21 ± 0.79^bc^	1.34 ± 0.17^bc^	86.83 ± 4.25^fgh^
MA15	23.38 ± 1.53^bcde^	12.17 ± 0.73^cd^	6.66 ± 0.55^bcde^	1.29 ± 0.08^bc^	82.50 ± 1.80^h^
IMC67	24.22 ± 1.17^abc^	11.55 ± 0.45^def^	6.76 ± 0.67^bcd^	1.28 ± 012^bcd^	85.83 ± 2.84^gh^
MO1	25.34 ± 1.74^ab^	12.11 ± 0.68^cde^	6.39 ± 0.65^bcdef^	1.23 ± 0.08^bcde^	92.33 ± 5.51^fgh^
BE10	23.64 ± 1.83^abcd^	12.33 ± 0.56^bcd^	6.90 ± 0.83^bcd^	1.37 ± 0.22^b^	85.33 ± 7.33^fgh^

*Note*: Results are expressed as mean ± standard deviation. Different letters within the same column indicate significant differences according to Tukey's test (*p* < 0.05).

The genotype CAB214 produced the smallest beans, with an average length of 19.95 ± 1.56 mm, thickness of 5.35 ± 0.58 mm, and weight of 0.67 ± 0.12 g. In contrast, genotype CCN51 showed the largest beans, with a length of 25.47 ± 2.00 mm, a width of 13.82 ± 0.99 mm, and an average weight of 1.72 ± 0.26 g.

The result obtained for CCN51 was expected, as this genotype is widely recognized for its superior agronomic traits, including high yield, disease resistance, and precocity. For these reasons, it is one of the most cultivated genotypes in countries such as Ecuador, Peru, and Brazil (Andrade et al. 2019). The larger bean size of CCN51 is also one of the factors that justify its extensive use as a parent in cocoa breeding and selection programs, reinforcing its strategic importance for global cocoa production (Jaimez et al. 2022).

The variability observed can be attributed to different factors, including moisture content, which influences linear dimensions and average weight (Tobias‐Baeza et al. 2024); shell content, which directly affects bean weight; and genetic variation (López‐Hernández et al. 2023). Despite these differences, the obtained characteristics are consistent with those previously reported for Forastero cocoa beans (Bart‐Plange and Baryeh 2003).

Phenotypic differences can influence fermentation time, drying efficiency, and susceptibility to pests and diseases (Kongor et al. 2016). Moreover, the number of beans per 100 g is a commercially relevant parameter, used by the Federation of Cocoa Commerce (FCC) as a classification criterion in four categories: standard beans (<100), medium beans (101–110), small beans (111–120), and very small beans (>120) (Akoa et al. 2021).

Among the Brazilian Amazonian genotypes, the bean count ranged from 69.5 ± 0.70 to 149.3 ± 0.58 beans/100 g, encompassing all classification categories. Eleven genotypes (CCN51, P7, CA6, PA121, PA195, CAB270, MA11, MA15, IMC67, MO1, and BE10) had values below 100 beans/100 g, thus classified as standard beans. Three genotypes (PA169, RB36, and CAB499) fell into the medium bean category (101–110), which is generally more valued commercially due to higher yields during roasting. Genotype CAB324 was classified as small beans (111–120), while RB40, CAB208, and CAB214 exceeded 120 beans/100 g, being categorized as very small beans, a trait often associated with lower cocoa mass yield and higher shell proportion.

#### 3.3.2 Cut Test

Biochemical and physical variations caused by fermentation can be assessed through the cut test, which involves making a longitudinal cut in the beans and classifying them based on cotyledon color and compartmentalization. Violet and poorly compartmentalized beans are considered unfermented, while brown or partially brown beans with compartmentalization are considered fermented (Lima et al. 2024).

**FIGURE 5:**
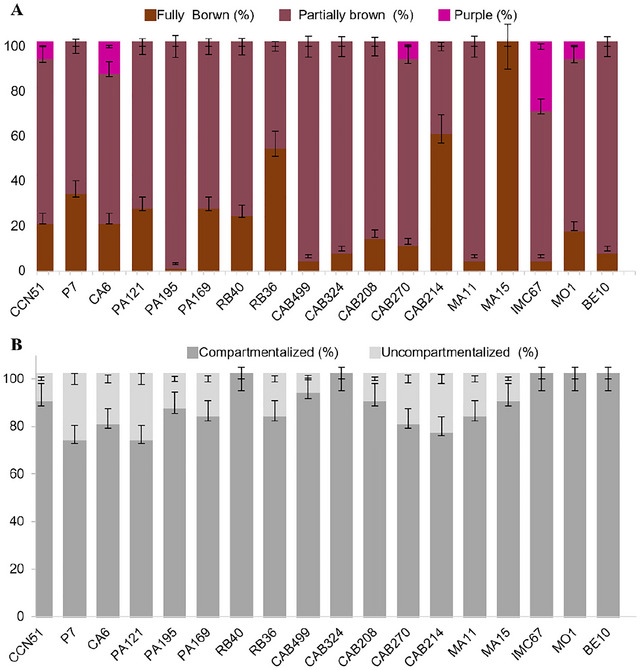
Cut test scores of cocoa beans from 18 Brazilian Amazon genotypes after starter culture‐induced fermentation (*Pichia kudriavzevii* LPB 06, *Lactiplantibacillus plantarum* L36, and *Acetobacter pasteurianus* A06) and drying: (A) cotyledon color; (B) cotyledon compartmentalization.

Despite differences observed in the cut test across the 18 Amazonian cocoa genotypes (Figure 5A), all showed a high proportion of fermented beans. Genotypes such as MA15 and CAB214 exhibited a higher percentage of fully brown beans (≥63%), whereas CAB499, CAB324, PA195, MA11, and BE10 showed predominance of partially brown beans (≥90%). In contrast, only genotype IMC67 displayed a significant proportion of violet beans (26.67 ± 5.77%). Regarding compartmentalization, all genotypes analyzed showed ≥75% compartmentalized beans (Figure 5B).

Ferreira et al. (2022) evaluated the impact of different induced fermentations, using inoculation with *Pichia manshurica* and *Saccharomyces cerevisiae*, on the color of cocoa beans through the cut test. Beans fermented with *S. cerevisiae* showed more than 85% well‐fermented beans (brown color), while the treatment with *P. manshurica* resulted in higher proportions of partially brown (10.67%) and purple beans (16.67%).

Biochemically, the brown coloration of beans indicates the degradation of phenolic compounds. During fermentation, anthocyanins are hydrolyzed into sugars and cyanidin, while phenolic compounds are oxidized into quinones by the action of polyphenol oxidase. Quinones, being highly reactive, interact with free amino acids and sulfhydryl groups to form melanoidins, high‐molecular‐weight brown pigments (Santander Muñoz et al. 2020). Cotyledon compartmentalization results from structural grooves caused by the diffusion of ethanol and acetic acid during fermentation (Lima et al. 2024).

Although bean size is often considered a relevant factor, it showed no significant correlation with fermentation degree according to the cut test (data not shown). For example, genotype MA15, with larger beans, displayed cut‐test results like CAB214, which produces smaller beans (Table 3). Moreover, among genotypes with higher proportions of purple beans, both small‐ and large‐sized beans were observed.

Another relevant factor for fermentation quality is the sample size used in induced fermentation. In general, fermentations with <15 kg of beans yield profiles similar to standard large‐scale fermentation (>40 kg) in terms of pulp temperature and pH of cotyledons, but they typically result in lower uniformity, with a higher proportion of partially brown and purple beans (Tunjung‐Sari et al. 2021).

### 3.4 Influence of Induced Fermentation on Different Genotypes

**FIGURE 6:**
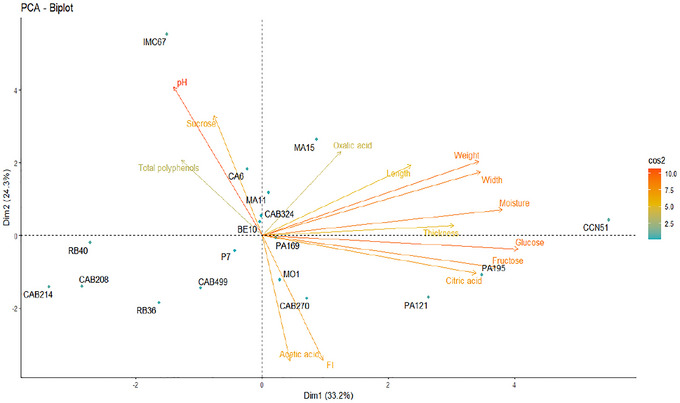
Biplot of the principal component analysis (PCA) of the physicochemical parameters of cocoa beans from 18 Brazilian Amazon genotypes after starter culture‐induced fermentation (*Pichia kudriavzevii* LPB 06, *Lactiplantibacillus plantarum* L36, and *Acetobacter pasteurianus* A06) and drying. The color gradient corresponds to cos² values, indicating the quality of variable representation on the PCA plane, with higher values reflecting stronger contributions to the principal components.

PCA was performed to reduce data dimensionality and evaluate the distribution of the 18 Brazilian Amazon cocoa genotypes based on the physicochemical characteristics of the cotyledons after induced fermentation. All analyzed variables were included in the PCA, except for the cut test, as it is a subjective analysis. A biplot of the first two principal components is shown in Figure 6, explaining 57.5% of the total data variability. The variables that most contributed to genotype separation along principal component 1 (PC1) were glucose, fructose, bean weight, width, and moisture, while pH and sucrose were the most relevant for principal component 2 (PC2).

The IMC67 genotype differed from the others by exhibiting the highest pH (5.85) and the residual sucrose content (2.46 mg/g DM). Elevated pH values (5.5–5.8) are indicative of incomplete fermentation, which may compromise cocoa quality (Voigt et al. 2018). Although the cut test was not included in the multivariate analysis, it also showed that IMC67 had the highest proportion of violet beans (Figure 5), reinforcing evidence of incomplete fermentation. Since most acidic invertases display optimal activity between pH 4.5 and 5.5 (Kongor et al. 2016), a pH above this range may have limited enzymatic efficiency, resulting in lower sucrose hydrolysis into glucose and fructose, and thus explaining the high residual sucrose levels observed in this genotype.

In contrast, CCN51 stood out for producing the largest beans, as well as the highest glucose and fructose concentrations. The availability of reducing sugars is essential as substrates for subsequent chemical reactions, particularly the Maillard reaction during roasting (Afoakwa et al. 2008).

PA195 and PA121 showed strong associations with glucose, fructose, and citric acid, which may directly influence the sensory properties of cocoa and derived products. CAB214, CAB208, and RB40 produced smaller beans with lower moisture content and similar physicochemical profiles. Genotypes positioned near the center of the PCA biplot displayed intermediate values for most variables, suggesting more balanced physicochemical characteristics.

The differences observed in the internal composition of the cocoa beans reflect not only the action of fermentative metabolites but also intrinsic genotype‐related characteristics, including structural properties of the beans that modulate compound diffusion. Recent evidence indicates that the testa exhibits genotype‐dependent thickness, permeability, and adsorptive capacity, thereby decisively influencing substance transport during fermentation (Camargo et al. 2024).

One of the main challenges when performing small‐scale laboratory fermentations is ensuring that each trial results in adequately fermented beans. Even under a standardized protocol, genetic differences among genotypes can significantly affect both the rate and extent of biochemical transformations during fermentation (Santander Muñoz et al. 2020). Thus, distinct fermentation dynamics across genotypes are expected.

## 4 Conclusion

Although induced small‐scale fermentation produced acceptable bean quality for most genotypes, it was insufficient to fully standardize their physicochemical profiles, underscoring the strong influence of genetic background on fermentation outcomes. Genotypes such as IMC67 may require extended fermentation times or optimized starter culture strategies to ensure complete fermentation. These findings highlight the importance of considering genetic diversity to optimize fermentation processes and implementing genotype‐specific or segregated management strategies to ensure greater consistency and uniformity in cocoa destined for the market. Furthermore, future studies should investigate additional factors influencing the chemical and sensory profiles of cocoa beans, including pulp chemistry, microbial metabolite dynamics, and the characterization of volatile compounds, to achieve a more comprehensive understanding of genotype–fermentation interactions.

## Author Contributions

**Giulia Victória Silva Lima**: writing – original draft, writing – review and editing, methodology, formal analysis, investigation, Data curation. **Anne Suellen Oliveira Pinto**: investigation, methodology, visualization. **Maria Glaucilene Dos Santos Correia**: investigation, methodology, visualization. **Marcos Paulo Meireles Filho**: investigation, writing – original draft, methodology, formal analysis. **Andre da Luz de Freitas**: investigation, formal analysis, writing – original draft, methodology. **Patrícia Oliveira Santos**: investigation, writing – original draft, formal analysis, methodology. **Hervé Rogez**: conceptualization, funding acquisition, writing – review and editing, project administration, supervision, resources.

## Conflicts of Interest

The authors declare no conflicts of interest.

## Supporting information




**Supplementary Material**: jfds70908‐sup‐0001‐Figure1.docx

## Data Availability

All data generated during this study are included in this published article.
