# Supplementary material for: Starter Culture‐Induced Fermentation Reveals Genotype‐Driven Variability in the Composition and Quality of Brazilian Amazon Forastero Cocoa Beans
Source: J Food Sci. 2026 Feb 13;91(2):e70908. doi: 10.1111/1750-3841.70908 (PMC12902724; doi:10.1111/1750-3841.70908)
Supplement: Supplementary file 1 — Supplementary Material: jfds70908‐sup‐0001‐Figure1.docx [file JFDS-91-0-s001.docx]

**
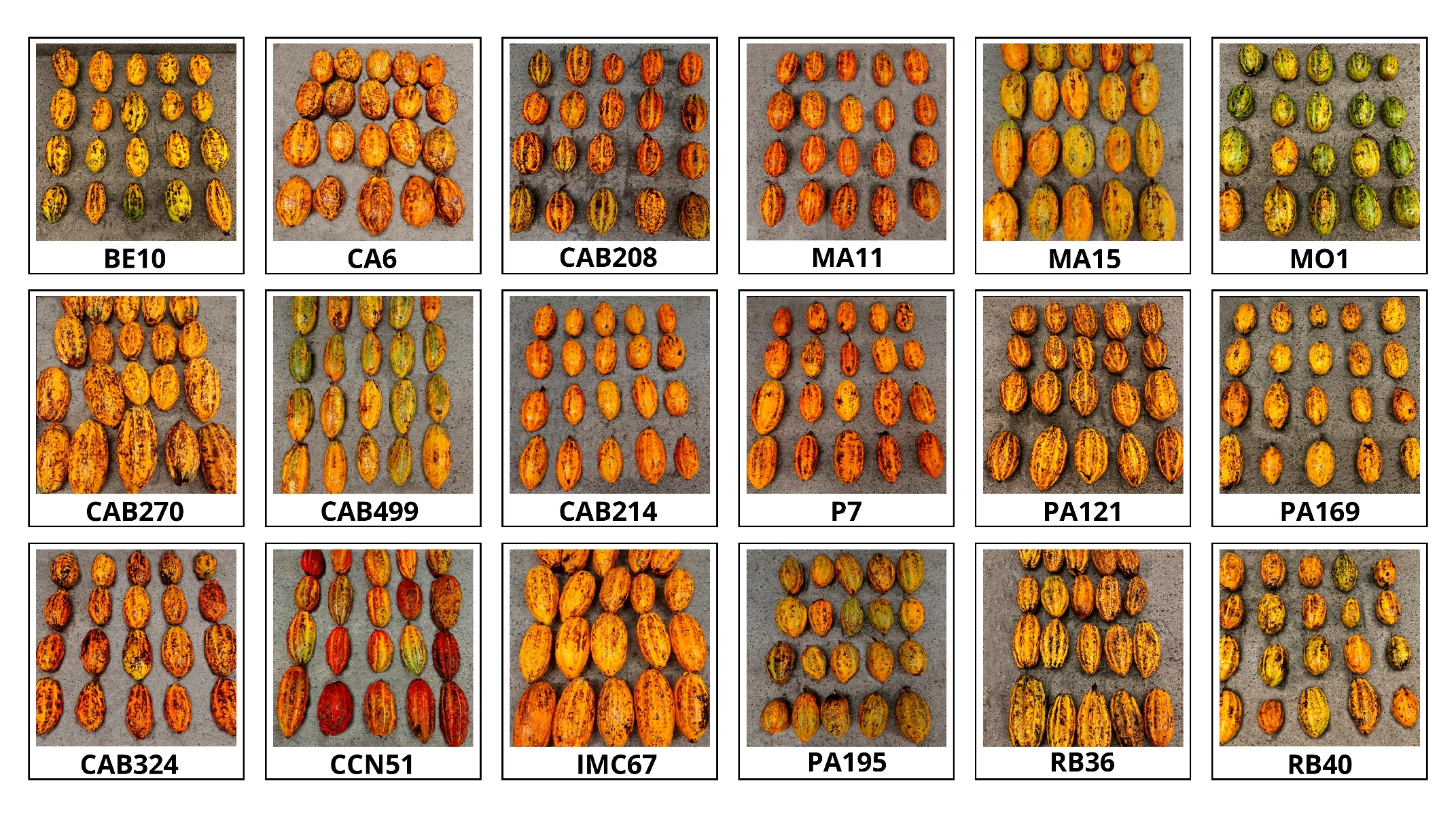
 Supplementary Figure 1.** Cocoa fruits from 18 genotypes of the germplasm bank of the Executive Commission of the Cocoa Farming Plan (CEPLAC), Pará, Brazil.
